# Influenza activity in Thailand and occurrence in different climates

**DOI:** 10.1186/s40064-015-1149-6

**Published:** 2015-07-16

**Authors:** Slinporn Prachayangprecha, Preeyaporn Vichaiwattana, Sumeth Korkong, Joshua A Felber, Yong Poovorawan

**Affiliations:** Department of Pediatrics, Faculty of Medicine, Center of Excellence in Clinical Virology, Chulalongkorn University, Bangkok, 10330 Thailand; Josef Korbel School of International Studies, University of Denver, Denver, CO 80210 USA

**Keywords:** Influenza surveillance, Influenza A, Influenza B, Influenza A(H1N1)pdm09, Influenza A(H3N2), Thailand

## Abstract

This study observed influenza activity between June 2009 and July 2014 in Thailand, a country in the Northern hemisphere with a tropical climate, and compared the results to activity in the United States (US) and Australia, which represent temperate climates in the Northern and Southern hemispheres, respectively. From Thailand, a total of 17,416 specimens were collected from patients exhibiting influenza-like illnesses and subjected to real-time PCR for the detection of influenza viruses. For comparison, laboratory confirmations of influenza originating from the US and Australia were obtained from the US CDC’s FluView surveillance reports and the Australian Government’s Department of Health and Ageing websites. We found that, generally, the influenza season in Thailand starts with the rainy season. This observation of influenza’s annual incidence pattern provides a better understanding of its occurrence, suggesting that vaccination campaigns should be started before the influenza season begins in order to reduce transmission.

## Background

In the last decade, the Kingdom of Thailand has rapidly evolved in terms of influenza control and pandemic influenza preparedness. Since 2004, these have been the explicit goals for a series of government-led initiatives aimed at reducing the burden of influenza in Thailand, both for the advancement of public health and the maintenance of national security. Thus, the Thai Ministry of Public Health (MOPH), in collaboration with the World Health Organization (WHO) and the United States Centers for Disease Control (CDC), has established itself at the forefront of global influenza surveillance and research. Now, after 10 years of national surveillance by two, independent studies, it is useful to review and analyze the data for the benefit of future public health policymaking in Thailand.

In 2004, the impetus for a national initiative on influenza control and pandemic preparedness was found when a new strain of highly pathogenic avian influenza A(H5N1) swept through Thailand, causing an estimated THB 25.24 billion in economic damages within its first 3 months (Tiensin et al. 2005). Between 2004 and 2006, Thailand recorded 3 more rounds of H5N1 outbreaks in humans before government interventions finally contained the problem by 2007 (The Second National Strategic Plan for Prevention and Control of Avian Influenza and Preparedness for Influenza Pandemic (B.E. 2551–2553) (A.D. 2008–2010) 2007). Nevertheless, avian and swine influenza viruses remain endemic among Thai fauna, where they circulate and may possibly recombine to form novel strains. Influenza A(H1N1)pdm09, for example, was a novel, reassortant strain estimated to have caused between 151,700 and 575,400 human deaths in its first 12 months, with over half occurring in Africa and Southeast Asia (Dawood et al. 2012). Based on these experiences and others, the government of Thailand is convinced that influenza represents a major threat to both public health and national security. Thus, since 2004, influenza has been the focus of public health interventions in Thailand.

Vaccination has proven to be the most effective strategy for preventing influenza transmission and reducing morbidity (CDC 2010). The WHO makes annual recommendations on the viral strains that should be vaccinated against for both the Northern and Southern hemispheres (Hampson 2008). Their recommendations are based on global influenza surveillance data, including genetic characterizations of circulating viruses, while the vaccines are based on the virion’s surface glycoproteins hemagglutinin (HA) and neuraminidase (NA) (Gerdil 2003). In 2004, the Thai MOPH introduced a national influenza surveillance system in cooperation with the US CDC (CDC 2012). The following year, in cooperation with the WHO, Thailand implemented its first National Strategic Plan for Avian Influenza Control and Pandemic Influenza Preparedness (National Strategic Plan for Avian Influenza control in Thailand (B.E. 2548–2550) (A.D. 2005–2007) 2005). Thanks to these initiatives, Thailand has brought its domestic influenza surveillance and vaccination programs up to the global standard.

During the 2009 influenza pandemic, the Center of Excellence in Clinical Virology of the Faculty of Medicine of Chulalongkorn University began to collect influenza surveillance data from four Thai provinces to better understand patterns of seasonality and typology of influenza in Thailand. We analyze the data in this study and compare it against national surveillance data from the US and Australia to assess the similarities and differences between influenza activity in different climates. We believe that this information is important for Thailand’s current influenza strategy and the future of public health policymaking in Thailand.

## Methods

### Geographic location

Thailand is a tropical country located between latitudes 5°37ʹN and 20°27′N and between longitudes 97°22′E and 105°37′E. Annual temperatures range between 0.8 and 44.5°C, with an average temperature of 27 degrees and an average relative humidity of 76% (Manisan 1995). The United States and Australia, on the other hand, are temperate countries located well within the Northern and Southern hemispheres, respectively.

### Specimen collection

**Figure 1:**
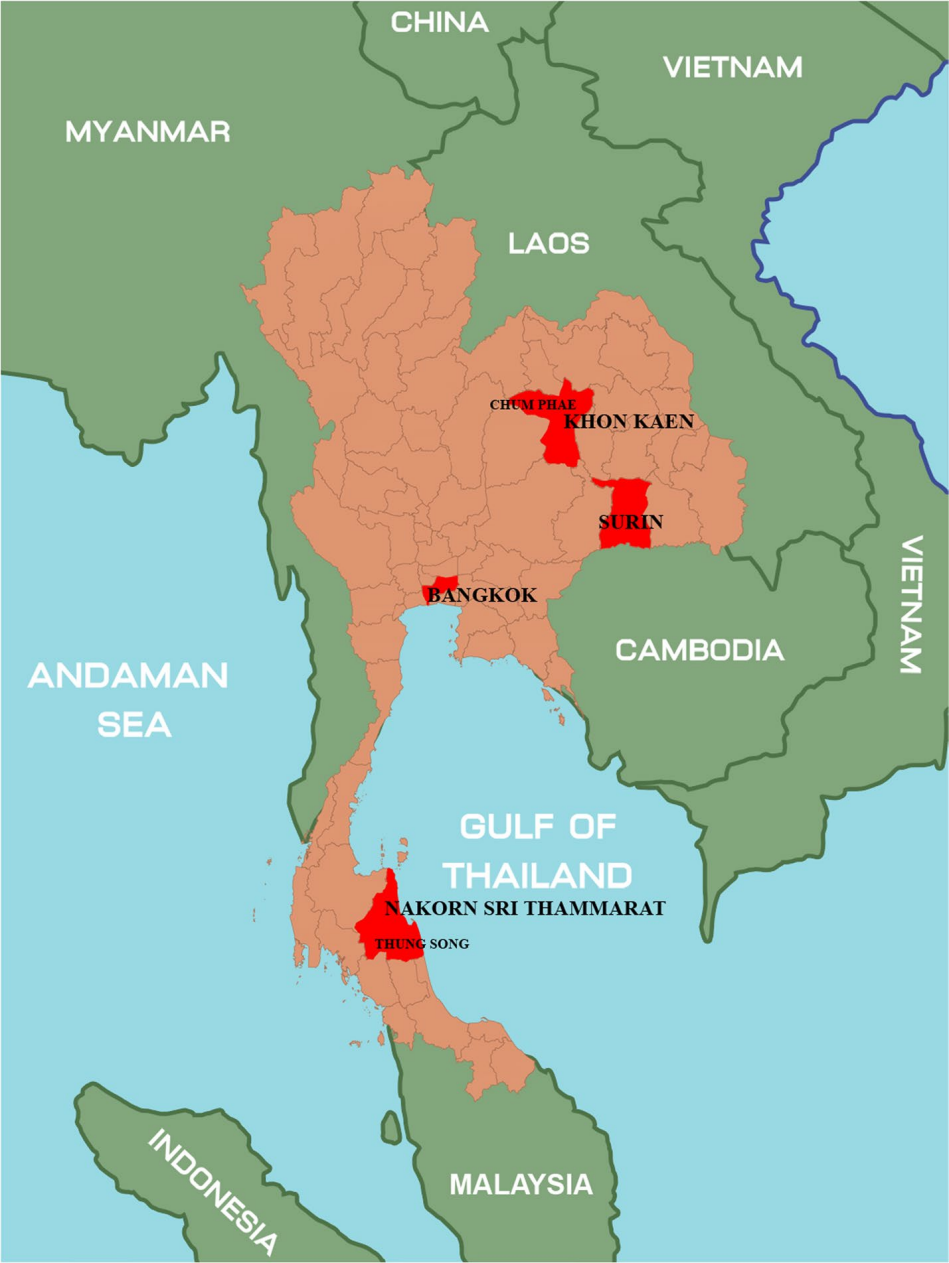
Location of specimen collection; Chum Phae, Khon Kaen provinces, Surin province, Bangkok and Thung Song, Nakorn Sri Thammarat province.

Between June 2009 and July 2014, 17,416 nasopharyngeal swab samples were taken during hospitalizations and outpatient services at geographically disparate hospitals in Thailand. Locations included Bangkok (n = 10,361), Chumphae district, Khon Kaen province (n = 5,736), Surin province (n = 119) and Thungsong district, Nakorn Sri Thammarat province (n = 1,200) (Figure 1). We did not analyze influenza activity in each hospital or province separately, as the goal of our surveillance was to determine activity based on the entire sample.

All specimens were collected individually from medically diagnosed cases of influenza-like illness (ILI), which was determined through the presence of high fever (>38°C) and respiratory tract symptoms. Participants were selected routinely based on these criteria alone and not differentiated by demographic factors such as age, sex, ethnicity, etc. Once collected, each sample was maintained at 4°C within a standard viral transport medium containing the antibiotics penicillin (G 2 × 106 U/L) and streptomycin (200 mg/L) and sent for laboratory analysis within 48 h to the Center of Excellence in Clinical Virology in Bangkok. Laboratory work involved subjecting each sample to RNA extraction and influenza virus detection through real-time RT-PCR (Suwannakarn et al. 2008; CDC protocol of realtime RTPCR for influenza A(H1N1) 2009). These procedures were conducted at the King Chulalongkorn Memorial Hospital by researchers from the Faculty of Medicine of Chulalongkorn University. Data for this study was obtained from previous reports (Prachayangprecha et al. 2010, 2011; Kanchana et al. 2012) and routine surveillance data acquired by the Center of Excellence in Clinical Virology, Faculty of Medicine, Chulalongkorn University. This research was carried out according to university’s ethical guidelines and was exempted by the Institutional Review Board of the Faculty of Medicine of Chulalongkorn University.

### Source of data for Influenza in the temperate zone

Cross-country comparison data were obtained from two sources chosen based on their reliability, quality, coverage and accessibility. To compare our influenza data to the northern hemisphere, we used the annual influenza reports from the United States provided by the US Centers for Disease Control and Prevention (CDC FLUVIEW interactive). To compare our influenza data to the southern hemisphere, we used the annual influenza reports from Australia provided by the Australian Department of Health and Ageing (Australian influenza report). We believe that these sources are ideal for comparison purposes because both collected data routinely from a large, nationally representative sample and published the data freely online. We further believe that the comparison is useful as both countries are geographically and demographically large in their respective hemispheres. As a medium-sized country located near the equator, Thailand should frequently examine similarities and differences between itself and both hemispheres.

## Results

### Influenza activity in Thailand

A total of 17,416 nasopharyngeal swab samples were collected from June 2009 to July 2014: 3,435 in 2009, 4,343 in 2010, 2,262 in 2011, 2,025 in 2012, 2,995 in 2013 and 2,356 in 2014. Through RT-PCR testing, 4,553 (26.1%) of the samples were found to be positive for influenza viruses. Of the positives, 3,723 (81.8%) tested positive for influenza A viruses while 830 (18.2%) tested positive for influenza B viruses. Of influenza A subtypes, A(H1N1)pdm09 contributed 2,458 (66.0%) of all influenza A positives while A(H3N2) accounted for 1,222 (32.8%). We also observed that seasonal, pre-pandemic A(H1N1) disappeared from our survey after 2011, though it was responsible for a small number of influenza A positives in 2009 and 2010.

In 2009, 1,318 of 3,435 (38.4%) samples were found to be influenza positive, with influenza A(H1N1)pdm09 accounting for 74.4% of all positive samples. Nevertheless, despite 2009 being the pandemic year, influenza B still accounted for 12.7% of positives. In 2010, 1,444 of 4,343 samples (33.2%) were tested positive for influenza viruses, with influenza A(H1N1)pdm09 responsible for 67% of all positives and influenza B, 21.1%. In 2011, 528 of 2,262 samples (23.3%) were confirmed for influenza and 66.5% of those were positive for influenza A(H3N2). Influenza B was responsible for 12.9%. In 2012, 346 of 2,025 samples (17.1%) were influenza positive, of which 45.7% were influenza B viruses and 54.3%, influenza A. Of the sub-typed influenza A viruses, 26.9% were influenza A(H1N1)pdm09 and 27.5% were influenza A(H3N2). In 2013, 410 of 2,995 samples (13.7%) were influenza positive, mostly for A(H3N2), which contributed 92.7% of all positives. In 2014, 507 of 2,356 samples (21.5%) tested positive, with influenza A(H1N1)pdm09 accounting for 57.8% of positives, while influenza B contributed 24.1%.

### Comparative influenza activity between tropical and temperate countries

**Figure 2:**
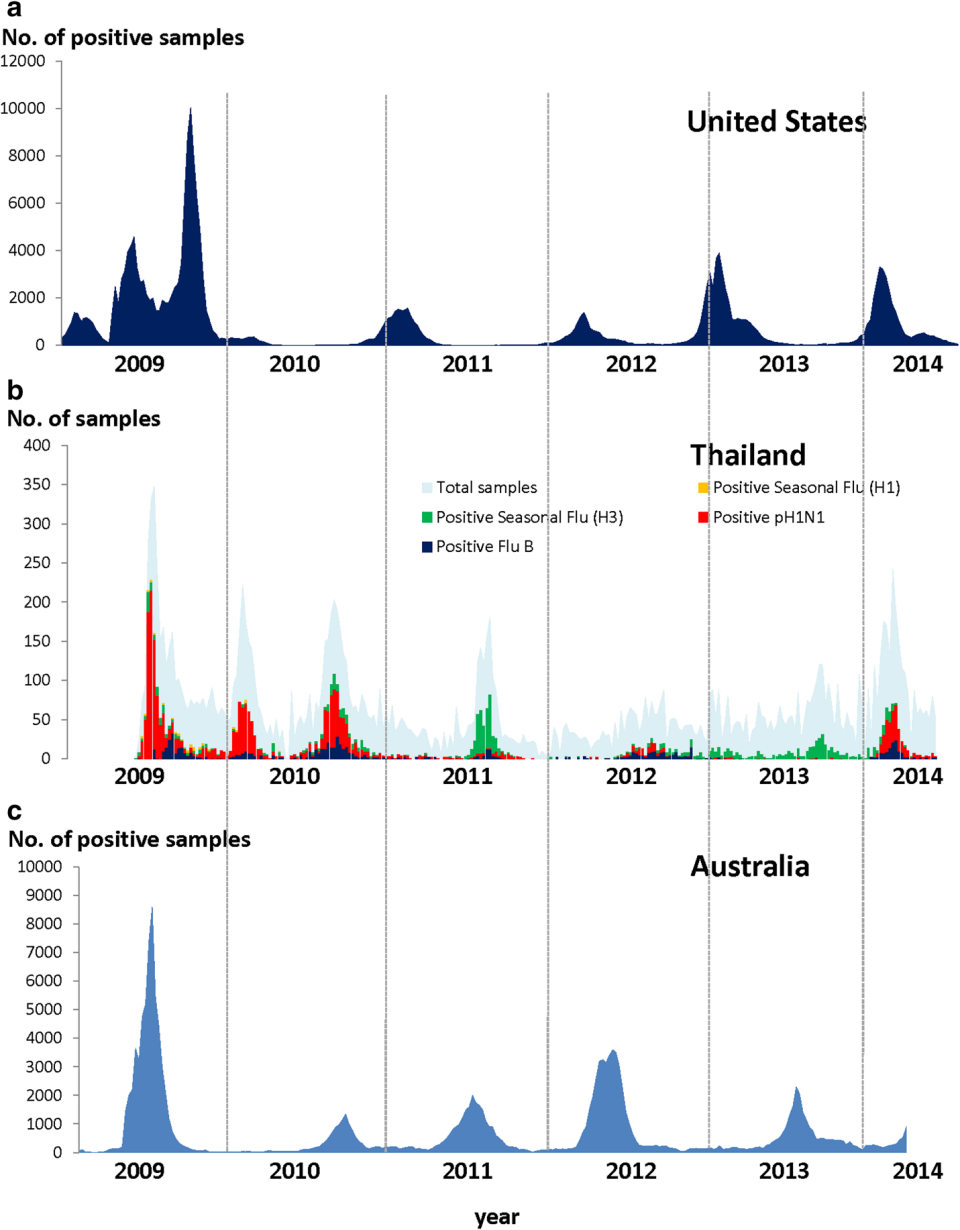
Weekly influenza activity during 2009–2014 influenza season. **a** United States, 2009–July 2014; **b** Thailand, July 2009–July 2014; **c** Australia, 2009–July 2014.

Figure 2 illustrates the number of specimens that were positive for influenza viruses from 2009 to 2014 in the United States, Australia and Thailand. The total number of patients per week with acute respiratory tract symptoms is also shown to compare against the cases of laboratory confirmed influenza viruses by type and subtype.

During the 2009 pandemic year, influenza surveillance in the US reported different periods of peak activity compared to Thailand and Australia. While the US experienced new infections almost continuously, Thailand and Australia showed only sporadic new cases just 3–4 months after the beginning of the outbreak. Furthermore, although the total number of new infections in the US was considered low in 2010, confirmed cases increased until the end of the year and continued into 2011. This is in contrast to Thailand, where influenza circulation produced two waves of infection, with the first wave lasting for a few months at the beginning of 2010 and the second occurring from August to September. Similarly, Australia recorded high levels of influenza activity from August to October.

During 2011, overall influenza activity in Thailand and Australia mirrored the previous year except that the curve began a few weeks earlier for Australia. In 2012, influenza activity in Thailand and Australia mirrored 2011, while in the US it started at the beginning of the year and lasted until June. In 2013, the US had a similar experience to 2012, but showed an increasing trend since early December, while Australia reported that the influenza season arrived later than in previous years. In Thailand, influenza activity remained at a low level. Between January and July of 2014, influenza activity in Thailand was similar to the US, beginning early in the year, and Australia’s influenza season started earlier than in 2013.

## Discussion

We analyzed differing patterns of influenza activity in the United States, Australia and Thailand. We chose to compare Thailand against the US and Australia because those countries represent temperate Northern and Southern hemisphere climates, respectively, while Thailand is a tropical Northern hemisphere country that is relatively close to the equator. In temperate regions, influenza activity has been associated with low temperatures and humidity (Tamerius et al. 2011), which explains why temperate countries typically experience influenza seasons during winter. In tropical climates, however, factors such as high temperatures, high humidity and high levels of precipitation are often linked to increased influenza activity (Chew et al. 1998; Yohannes et al. 2004; Mardy et al. 2009; Moura 2010). In fact, year-round influenza activity is more common in countries with tropical influenza seasonality (Viboud et al. 2006). Nevertheless, whether influenza activity will be particularly high or low in each season is still hard to predict due to the influence of many variables, including host factors, environmental factors and the virulence and/or pathogenicity of each strain (Tamerius et al. 2011; Tscherne and Garcia-Sastre 2011).

Our study has shown that Thailand’s annual influenza activity during our period of observation is more similar to Australia than the US, despite Thailand being located in the Northern hemisphere. We found that influenza activity in Thailand usually presents as an annual cycle, except during the 2009 pandemic and its aftermath, when the circulation of influenza A(H1N1)pdm09 caused the normal pattern to break. Most likely this was the result of the Thai population’s lack of immunity toward the new pandemic strain. Although in normal years there is a pattern of influenza seasonality in Thailand, except in 2012 and 2013, which we cannot clearly see the distinct cyclic peak like previous years because there were lower total ILI cases during 2012–2013, low influenza activity can still be detected outside of influenza seasons. For countries with smaller populations, such as Thailand and Australia, detecting influenza activity year-round may be more difficult than in larger countries like the US. Therefore, an increase in the number of testing specimens would likely improve influenza detection outside of influenza seasons (Kelly et al. 2013).

Interestingly, our data shows that the percentage of influenza positives obtained from our sampling of medically diagnosed cases of influenza-like illness has been decreasing over time. Except in 2014, for which we only have 7 months of data, the percentage of influenza positives decreased each year since 2009. This could also be an indication that other pathogens are becoming more important over time in causing influenza-like illnesses.

Thailand has three seasons: summer from February to May, the rainy season from June to September and the cool season from October to January. Influenza activity in Thailand tends to increase during the rainy season, which is typical for countries located in tropical latitudes (Agrawal et al. 2010; Nguyen et al. 2007). Thus, in order to effectively reduce annual influenza transmission, seasonal influenza vaccination campaigns should be started before late summer, prior to the influenza season. Nevertheless, it is still beneficial for people to be vaccinated during the influenza season. Another method to effectively reduce overall influenza transmission is to immunize children and young adults who experience the highest risk of contracting the virus due to personal hygiene and group activities involving close contact (Manzoli et al. 2007; Longini and Halloran 2005; Chieochansin et al. 2009). Because most schools in Thailand open in May, vaccinations usually start from April to May and may reduce influenza-related morbidity and mortality. Vaccination is also recommended for sub-populations that are especially at risk, such as children under 5 years of age, adults aged 65 years and over, pregnant women, healthcare workers and people with underlying health conditions such as chronic respiratory disease, chronic heart disease and obesity (WHO 2013). Annually, the WHO makes recommendations on influenza vaccine compositions for both the Northern and Southern hemispheres, with the chosen strains that are the good match with circulating strains. Vaccine production according to WHO guidelines for the southern hemisphere is usually conducted in time to be distributed just as the influenza season is starting in Thailand. However, it is important to remember that, even if there is some difference in the season’s timing or if the vaccine supply does not meet peak demand, the best vaccine should always be the available one, regardless of which hemisphere it is recommended for, as it can still be beneficial for influenza prevention.

Influenza activity that tends to peak during the rainy season, which is typical for many other tropical countries. With Thailand’s first domestic vaccine production facility to be completed in the near future, we believe that it is valuable to report on current surveillance data and influenza trends so that the operations of this factory can be planned for maximum cost-effectiveness. Aside from closely monitoring influenza activity, seasonal influenza vaccination with southern hemisphere strains is recommended for certain high-risk groups before the influenza season begins as part of a strategic vaccination program.
